# Developing body estimation in adolescence is associated with neural regions that support self-concept

**DOI:** 10.1093/scan/nsae042

**Published:** 2024-06-21

**Authors:** Yara J Toenders, Hannah Dorsman, Renske van der Cruijsen, Eveline A Crone

**Affiliations:** Developmental and Educational Psychology, Leiden University, Leiden 2333AK, The Netherlands; Leiden Institute for Brain and Cognition, Leiden University, Leiden 2333AK, The Netherlands; Erasmus School of Social and Behavioral Sciences, Erasmus University Rotterdam, Rotterdam 3062PA, The Netherlands; Erasmus School of Social and Behavioral Sciences, Erasmus University Rotterdam, Rotterdam 3062PA, The Netherlands; Behavioral Science Institute, Radboud University Nijmegen, Nijmegen 6500HE, The Netherlands; Developmental and Educational Psychology, Leiden University, Leiden 2333AK, The Netherlands; Leiden Institute for Brain and Cognition, Leiden University, Leiden 2333AK, The Netherlands; Erasmus School of Social and Behavioral Sciences, Erasmus University Rotterdam, Rotterdam 3062PA, The Netherlands

**Keywords:** adolescence, body image, self-concept, fMRI, body satisfaction

## Abstract

Both self-concept, the evaluation of who you are, and the physical body undergo changes throughout adolescence. These two processes might affect the development of body image, a complex construct that comprises one’s thoughts, feelings, and perception of one’s body. This study aims to better understand the development of body image in relation to self-concept development and its neural correlates. Adolescents (aged 11–24) from the longitudinal Leiden Self-Concept study were followed for three consecutive years (N_T1_ = 160, N_T2_ = 151, and N_T3_ = 144). Their body image was measured using a figure rating scale and body dissatisfaction questionnaire. Body estimation was calculated based on figure ratings relative to their actual body mass index (BMI). Additionally, participants evaluated their physical appearance traits in an functional magnetic resonance imaging (fMRI) task. Results revealed that body estimation and body dissatisfaction increased with age. Heightened inferior parietal lobe (IPL) activation during physical self-evaluation was associated with lower body estimation, meaning that the neural network involved in thinking about one’s physical traits is more active for individuals who perceive themselves as larger than they are. IPL activity showed continued development during adolescence, suggesting an interaction between neural development and body perception. These findings highlight the complex interplay between affective, perceptual, and biological factors in shaping body image.

## Adolescent development of body image: an fMRI study

Adolescence, the period between childhood and adulthood, is a formative developmental period during which self-concept changes (Sebastian et al. 2008, Crone et al. 2022). Self-concept is defined as the knowledge and beliefs that one has about oneself. During adolescence, when peers become increasingly important, peer opinions become influential in shaping the self-concept (Harter 2012, Jankowski et al. 2014). Additionally, during this developmental period, adolescents’ perspective taking skills increase, which contribute to the increased awareness of social evaluations (Blakemore 2012). That is, adolescents become more self-conscious and perceptive of others’ opinions (Blakemore 2012, Somerville 2013, Parker et al. 2015).

During adolescence, the body also undergoes physical changes, as it is growing and developing into an adult shape (Marshall and Tanner 1968). During this period, height and weight increase and secondary sex characteristics develop (De Ridder et al. 1992, Traggiai and Stanhope 2003). Additionally, an increase in fat tissue takes place during early puberty, with different fat patterning for males and females (Taylor et al. 2010). Both the changes in the body and in self-concept might affect body image. Body image is a complex construct that goes beyond the simple representation of appearance. It comprises a person’s thoughts, feelings, and perception of the aesthetics or sexual attractiveness of their own body (Grogan 2021). Prior studies have defined body image as a combination of multiple components including a subjective perceptual component (i.e. the experience of one’s body size) and a subjective affective component (i.e. the emotional attitudes towards one’s body) (Skrzypek et al. 2001).

It is currently now yet known how one’s body image is related to one’s broader self-concept. Possibly, body image is influenced by one’s physical self-concept (Mendo-Lázaro et al. 2017). Even though self-concept generally develops throughout adolescence, its development is domain dependent (van der Cruijsen et al. 2018). The perceptual component of body image is thought to increase with age, as prior research showed that children aged 6 years old overestimate their body size, which becomes more accurate towards mid-adolescence (Gardner 2012). Body dissatisfaction, an evaluative component of body image, also increases throughout adolescence, and stabilizes towards adulthood (Eisenberg et al. 2006, Wang et al. 2019). Research has shown that children with lower body dissatisfaction also showed a more positive physical self-concept. Additionally, females experience higher levels of body dissatisfaction than males during adolescence (Kantanista et al. 2015). Body image is negatively influenced by one’s objective body size [e.g. body mass index (BMI)], suggesting that most adolescents strive for a thin body ideal (Streeter et al. 2012). Despite the importance of a healthy body image in adolescence, the relation between the physical self-concept development and perceptual and affective body image remains unknown.

One way to understand the relation between self-concept development and body image changes is by studying the neural correlates of evaluating physical traits. Prior functional magnetic resonance imaging (fMRI) studies examining the neural underpinnings of self-concept have posited three brain networks implicated in self-concept (Crone et al., 2022, Pfeifer and Peake 2012). The first network is responsible for autobiographical memories and encompasses midline structures of the medial prefrontal cortex (mPFC) and parietal cortex. The second network is involved in social-cognitive aspects of self-concept, to which the ventral mPFC contributes. Lastly, a third network is engaged in perspective taking, for which the temporal parietal junction (TPJ) and superior temporal sulcus (STS) are crucial. In previous research (Pfeifer et al. 2009, 2013, van der Cruijsen et al. 2018, 2023), participants were asked to rate traits as applicable to themselves in three domains: academic, prosocial, and physical (i.e. appearance). This research has shown that various domains of self-concept (academic, prosocial, and physical) exhibit distinctive neural correlates (van der Cruijsen et al. 2017, 2018). Increased neural activity in the ventrolateral prefrontal cortex (VLPFC), inferior parietal lobe (IPL), inferior temporal gyrus (ITG), precuneus/posterior cingulate cortex (PC/PCC), and dorsomedial prefrontal cortex (dmPFC) was found in healthy adolescents when they evaluated themselves on physical traits (van der Cruijsen et al. 2018), relative to academic and prosocial traits.

Self-concept development in relation to body image development has not yet been investigated in normative adolescent samples. However, task-based fMRI studies in a clinical sample of patients with Anorexia Nervosa, a body image distortion disorder that often has its onset in adolescence (Micali et al. 2013), have found similar regions to be involved in body image when participants were asked to evaluate their physical traits. These studies suggested that prefrontal and insular cortices and the amygdala are involved in processing the affective component of body image, while posterior parietal areas such as the IPL are implicated in processing the perceptual component (Wagner et al. 2003, Uher et al. 2005, Sachdev et al. 2008, Vocks et al. 2010a). While a substantial body of research has examined the neural correlates of body image in a clinical population diagnosed with Anorexia Nervosa, it is not yet well understood how these neural regions develop over time in typically developing adolescents. Since body image is part of the physical self-concept construct, the neural correlates of physical self-concept could provide further information on the development of body image in adolescence.

Given the importance of a positive body image for wellbeing (Tylka 2011) and the limited understanding of the relation between body image and self-concept development, the current study aimed to investigate the development of body image and its neural correlates in adolescents using the physical self-concept task previously published by van der Cruijsen et al. (2018, 2023). In this accelerated longitudinal fMRI study, adolescents aged 11–21 years old were followed for three consecutive years to study self-concept development from a brain-behavior perspective. All participants also completed objective [body mass index (BMI)], perceptual subjective [body estimation defined as the alignment between image of own body (on the figure rating scale) and actual BMI], and affective subjective (body dissatisfaction and the difference between own and ideal body on figure rating scale) measures of body images at all timepoints. It was hypothesized that BMI increased throughout adolescence, and that body dissatisfaction (affective body image) would increase throughout adolescence (Marshall and Tanner 1968, Eisenberg et al. 2006, Wang et al. 2019). Based on these two measures, it was expected that the perceptual measure of body image (body estimation) becomes more accurate with age (Gardner 2012). We additionally studied the association between body image and physical self-concept to investigate whether body image and physical self-concept were associated. We hypothesized that body image was associated with increased neural activity in brain regions associated with physical self-concept (van der Cruijsen et al. 2018, 2023) and body image processing (Vocks et al. 2010a), such as prefrontal and insular cortices and posterior parietal areas. Finally, we examined how neural activation developed and if there is a longitudinal association between body image and neural activity during evaluating the physical self.

## Methods

### Participants

Data from the longitudinal Leiden Self-Concept study were used. This study used an accelerated design with three timepoints that consisted of a lab visit where behavioral and fMRI data were collected. The sample included 160 participants aged between 11 and 21 at the first timepoint. At the second and third timepoints, these participants were again asked to participate in the study, and new participants were invited too (N at T2 = 151, N at T3 = 144). Demographic characteristics can be found in Table 1. The inclusion criteria were right-handedness and normal or corrected-to-normal vision. Exclusion criteria were MRI contraindications, psychotropic medication usage, and psychiatric or neurological disorder diagnosis. Written informed consent was provided by all participants and parental consent for participants below 18 years old. This study was approved by the Medical Ethics Committee of the Leiden University Medical Centre. Each MRI scan was reviewed by a radiologist and no clinically relevant findings were observed.

**Table 1.** Demographics at T1. Mean and SD are displayed.

Variables	Females	Males	Total
***N* (%)**	86 (53.8%)	74 (46.2%)	160
**Age**	15.93 (3.00)	15.91 (2.95)	15.92 (2.97)
**BMI**	20.41 (3.03)	19.83 (3.10)	20.14 (3.07)
**Education**			
Primary school	13 (15.1%)	17 (23.0%)	30 (18.8%)
High school	48 (55.8%)	40 (54.1%)	88 (55.0%)
Higher education	25 (29.1%)	17 (23.0%)	42 (26.3%)
**Body rating**	3.92 (1.22)	3.62 (1.21)	3.78 (1.22)
**Ideal body rating**	3.21 (0.93)	3.56 (0.97)	3.38 (0.96)
**Ideal body difference**	0.71 (1.15)	0.05 (1.03)	0.41 (1.14)
**Body estimation**	0.00 (2.15)	0.00 (2.40)	0.00 (2.27)
**Body dissatisfaction**	30.69 (10.22)	24.77 (9.24)	27.95 (10.19)
**Positive physical self-concept**	2.84 (0.50)	2.90 (0.49)	2.87 (0.50)
**Negative physical self-concept**	1.74 (0.51)	1.62 (0.41)	1.68 (0.47)

### Measures

#### Body image

Body image was assessed using objective and subjective measures. In Table 1, we present the average ratings and SD for each of these measures.

Objective body image, as indicated by participants’ BMI, was determined using the Quetelet’s index for calculating BMI with their measured weight and the square of height (Garrow and Webster 1985).

**Figure 1.**
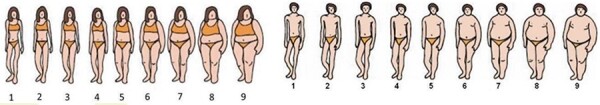
Figure rating scale. Participants had to indicate what image their body resembled most and what their ideal body looked like.

Subjective body image was measured in two ways: the perceptual and affective component. For these measures, a Figure Rating Scale, which represented body sizes among a scale from small to large, was used for boys and girls separately (see Fig. 1). Participants were asked what image they believed their body resembled most (“body rating”). Next, they were asked which rating resembled their ideal body (“ideal body rating”).

**Figure 2.**
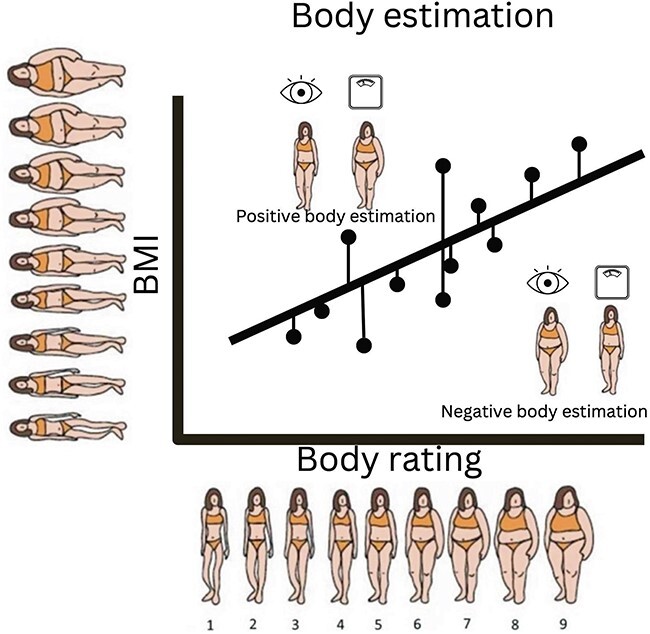
Body estimation is defined as the deviation from the body rating that is on average associated with a certain BMI. Participants had to indicate what image their body resembled most, and body estimation was calculated from the alignment between this rating and their actual BMI. The eye icon indicates how people evaluate their body and the scale icon indicates what the objective BMI was. Participants with a positive body estimation (above the regression line) evaluated their bodies as smaller than they are, whereas those with a negative body estimation (below the regression line) evaluated their bodies as larger.

The perceptual body image, the extent to which participants were able to assess their actual body size relative to their BMI, was measured using a “body size estimation” rating, reflecting the alignment between BMI and subjective body ratings on the Figure Rating Scale. That is, body size estimations were calculated as the residuals (i.e. deviations) from the linear regression where BMI was regressed on the body rating (Fig. 2). This resulted in standardized residual scores, which were used as a measure of body size estimation. Thus, body estimation is defined as the extent to which one’s body image aligns with the average body image for a specific BMI.

Individuals with a body size estimation of 0 evaluated their bodies as the average rating for their BMI, whereas those with a positive body size estimation have a higher BMI than the average BMI with that body image. In other words, one sees themselves as smaller than they are. Those with a negative body size estimation have a lower BMI than the average BMI with this body image, meaning that one sees themselves as larger than they are. This process was done separately for males and females.

The affective body image was calculated as the difference between the two responses on the Figure Rating Scale (body rating—ideal body rating) (“ideal body difference”). In addition to the Figure Rating Scale, subjective affective body dissatisfaction was measured using the body dissatisfaction subscale of the Eating Disorder Inventory-3 (EDI-3) (Garner 2004). This subscale assesses the dissatisfaction about different body parts. It consists of 13 items [e.g. ‘I feel satisfied with the shape of my body’ (reverse coded), ‘I think that my stomach is too big’’] and each item is graded on a 6-point Likert scale.

#### fMRI task description

The participants performed a self-concept task in the MRI scanner to detect neural activity related to physical self-concept (van der Cruijsen et al. 2018). In the task, participants were presented short sentences describing traits in the physical, academic, or prosocial domain (e.g. “I am (un)attractive”). These traits were either positively or negatively valenced. Participants were asked to indicate to what extent they believe a certain trait fits them on a scale from 1 (“not at all”) to 4 (“completely”). For each domain, 20 trait sentences were presented (10 positively valanced and 10 negatively valanced; see Supplementary Table S1 listing all items). Additionally, participants were presented control sentences. In the control condition, participants were shown 20 trait sentences and instead of rating whether this fit the participant, they had to categorize the traits into one of the following four categories: (i) school, (ii) social, (iii) appearance, or (iv) I don’t know. See Table 1 for the average ratings and the SD.

**Figure 3.**
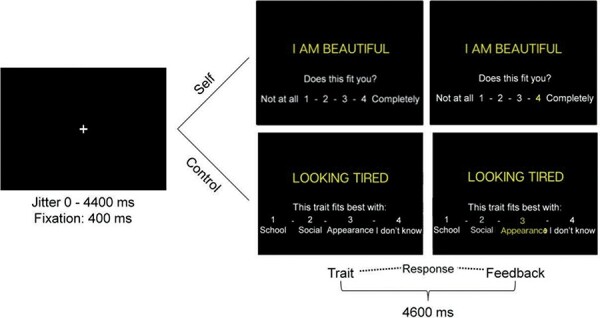
Trial examples of the physical self-concept and control condition of the self-concept task (adapted from van der Cruijsen et al. 2018).

The conditions occurred in separate runs and the order of the two conditions (self-concept and control) was counter balanced. Trials were presented in a pseudorandomized order. The participants were first presented with a fixation cross for 400 ms. Next, the stimulus (trait sentence with the answer options) was shown for 4600 ms, providing the participants with enough time to answer (Fig. 3). When they provided their answer, the number they chose turned yellow. If the participant did not answer within the allowed 4600 milliseconds, they were shown the “Too late!” message for 1000 milliseconds. These late trials were excluded from the analysis and were modeled independently. OptSeq was used to add jittered intertrial intervals, which varied between 0 and 4.4 s (Dale 1999). Given the focus of the current study, only the physical domain and control condition were examined.

#### fMRI data acquisition

MRI scans were acquired using a Philips Ingenia 3.0 Tesla MRI scanner with a standard whole-head coil. A self-concept fMRI task and a resting-state fMRI scan were obtained, followed by a structural MRI scan. Participants viewed the trait sentences on the screen behind the scanner using a mirror fastened to the head coil. The coil was fitted with foam inserts to limit head movement. T2*-weighted echo-planar imaging (EPI) sequence was used to collect functional images over the course of two runs [time repetition (TR) = 2200 msec, time echo (TE) = 30 msec, sequential acquisition, 37 slices of 2.75 mm, the field of view (FOV) = 220 × 220 × 111.65 mm]. To account for T1 saturation, the first two volumes were neglected. Next, a high-resolution 3D T1-FFE scan was acquired for registration purposes (TR = shortest msec, TE = 4.6 msec, 140 slices, voxel size = 0.875 mm, FOV = 224 × 178.5 × 168 mm).

#### fMRI preprocessing and statistical analysis

The fMRI data were analyzed using SPM12 (Wellcome Department of Cognitive Neurology, London). The images were corrected for slice-timing and rigid body movement. Next, they were normalized to T1 images using a 12-parameter affine transformation with nonlinear transformation involving cosine basis functions. Images were resampled to 3 mm in MNI stereotaxic space. Lastly, they were smoothed with a 6-mm FWHM gaussian kernel.

As reported in a prior study, general linear models in SPM12 were used to estimate task effects (van der Cruijsen et al. 2018). The timeseries were modeled as zero duration events convolved with the hemodynamic response functions (HRF). For the self-concept run, “Physical” was modeled as an event of interest, and in the control task, “Control” was modeled as an event of interest. In the general linear model, these events were added as covariates, together with cosine functions to high-pass filter the data. Additionally, six motion regressors were added to the model.

The contrast images were subsequently used for a group-level analysis. In the first analysis, the physical self-concept trials were compared to control trials in a one-sample t-test for the contrast Physical > Control. To examine the relation between neural responses to physical self-concept and body image evaluations in T1 data, three whole-brain regressions were performed for ideal body difference scores (based on the Figure Rating Scale), body dissatisfaction (based on the EDI), and body estimation (based on residual scores).

To further visualize significant relations, the Marsbar region of interest (ROI) toolbox was used to extract parameter estimates.

#### Data analyses

General linear mixed models were used to study the development of body image (separately for body rating, ideal body rating, perceptual body image: body estimation and affective body image: ideal body difference and body dissatisfaction) throughout development, with a random intercept for subject to control for nesting within-person. Additionally, it was tested whether adding a random slope improved model fit. Since this did not improve model fit, these models were not reported. Linear and quadratic associations with age were studied, with sex as a covariate. In addition, general linear models were also used to study the development of behavioral physical self-concept. The results were corrected for multiple comparisons using a Bonferroni correction per research question (i.e. I. developmental effects of body image, II. developmental effects of physical self-concept, and III. association between body image and physical self-concept). Lastly, the fMRI analyses, as described above, were executed. The association between neural activity and body image at T1 was examined using a general linear model, and this was repeated for T2 and T3. Additionally, it was examined whether neural activity at T1 preceded body image at T2 and T3 using general linear models.

## Results

**Figure 4.**
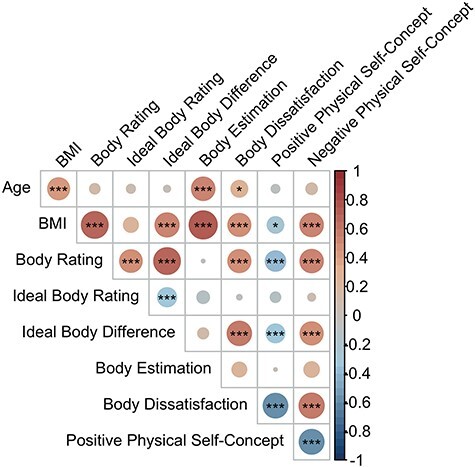
Correlation matrix displaying the correlation between body image measures and self-concept at T1. * *P *< .05, ** *P *< .01, *** *P *< .001. *P*-values are Bonferroni corrected.

Correlation between the variables on the first timepoint can be found in Fig. 4 (see Supplemental Figs. S1 and S2 for similar results for timepoints 2 and 3). Higher BMI correlated positively with a larger body image (“rating”), higher body estimation (estimating oneself as smaller than they are based on BMI), higher ideal body difference, higher body dissatisfaction, and more negative physical self-concept. Higher ideal body difference and higher body dissatisfaction correlated negatively with physical self-concept. Partial correlation analyses correcting for age did not change the results (see Supplemental Fig. S3).

### Development of body image

The development of the five measures of body image (body rating, ideal body rating, ideal body difference, body estimation, and body dissatisfaction) was first examined using general linear mixed models with age as linear and quadratic predictor and gender as between-subjects variable. The results show that body rating and ideal body rating were not associated with age (*B *= 0.01, *p*_bonferroni_ = 0.99; *B *= –0.01, *p*_bonferroni_ = 0.99).

Body estimation (the alignment between the perceived image of the self and BMI) showed a quadratic association with age (*B *= –.03, *p*_bonferroni_ = 0.01), also after correcting for BMI. This result shows that younger participants rated their bodies as larger relative to their BMI, while it flattened around zero for older adolescents.

**Figure 5.**
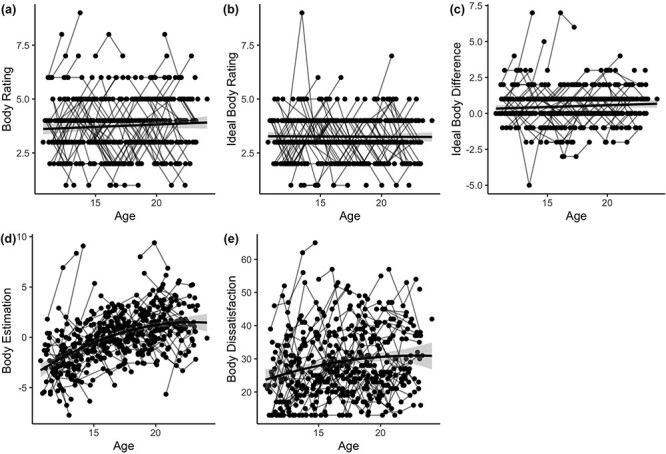
Development of body image. Displayed are the developments of five measures of body image (a: body rating, b: ideal body rating, c: ideal body difference, d: body estimation, e: body dissatisfaction). Body rating, ideal body rating, and ideal body difference were not associated with age. Body estimation and body satisfaction showed a quadratic association with age.

Ideal body difference (measured by the difference in rating of one’s own body and ideal body) was not associated with age (*B *= 0.04, *p*_bonferroni_ =0.32). In general, ideal body difference was significantly different from 0 (*t *= 4.29, *P *< .001), meaning that participants’ ideal body (3.35, SD: 0.97) was smaller than their own body (3.75, SD: 1.22). Lastly, body dissatisfaction as measured using the EDI gave a different result. This measure showed a quadratic association with age (*B *= –.12, *p*_bonferroni_ = 0.008; Fig. 5), showing that participants in mid-adolescence were more dissatisfied about their body than younger participants and this flattened in late adolescence.

### Physical self-concept—behavioral results

**Figure 6.**
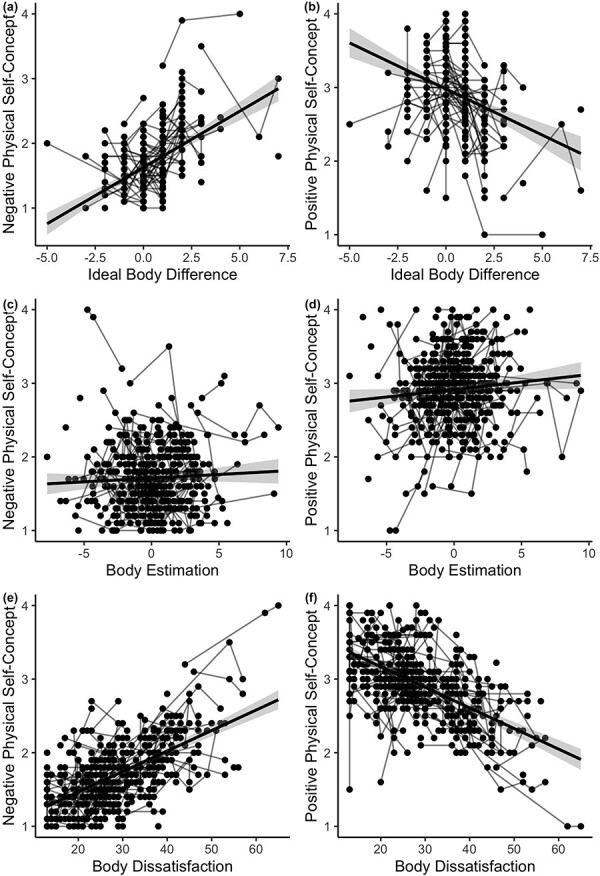
Correlation between body image and self-concept at T1. The relation between negative and positive physical self-concept and three measures of body image are displayed (a & b: ideal body difference, c & d: body estimation, e & f: body dissatisfaction). Ideal body difference and body dissatisfaction were associated with negative and positive physical self-concept (B = –0.56, *p*_bonferroni_ <0.001; B = 0.91, *p*_bonferroni_ <0.001; B = –9.25, *p*_bonferroni_ <0.001; B = 11.41, *p*_bonferroni_ <0.001). Body estimation showed an association with positive but not negative physical self-concept (B = 0.64, *p*_bonferroni_ =0.02; B = 0.03, *p*_bonferroni_ =0.99).

The same general linear mixed models with age as linear and quadratic predictor and gender as between-subjects variable were performed for self-concept ratings. Negative and positive physical self-concept did not show a linear or quadratic association with age (B = 0.02, *p*_bonferroni_ = 0.05; B = 0.004, *p*_bonferroni_ = 0.19; B = 0.003, *p*_bonferroni_ = 0.99; B = –.003, *p*_bonferroni_ = 0.30). To examine the relation between the wider construct of physical self-concept and body image, relations with the body image measures are displayed in Fig. 6.

### Physical self-concept—fMRI results

As presented in previous research using the same sample (van der Cruijsen et al. 2018), a whole-brain one-sample t-test for the contrast Physical self-concept > Control was used to examine brain activity during self-concept evaluation at T1. This analysis showed significantly higher activation during the physical self-concept condition than the control condition in network of regions including the vlPFC (x = 44, y = 40, z = 12, cluster size = 309; x = 28, y = 32, z = –14, cluster size = 50; x = 20, y = 46 z = 30, cluster size = 68; x = –24, y = 48, z = 30, cluster size = 73; x = –28, y = 36, z = –12, cluster size = 24), dmPFC (x = 2, y = 58, z = 12, cluster size = 1534), and IPL (x = 54, y = –32, z = 46, cluster size = 288).

### Body image and physical self-concept—fMRI results

**Figure 7.**
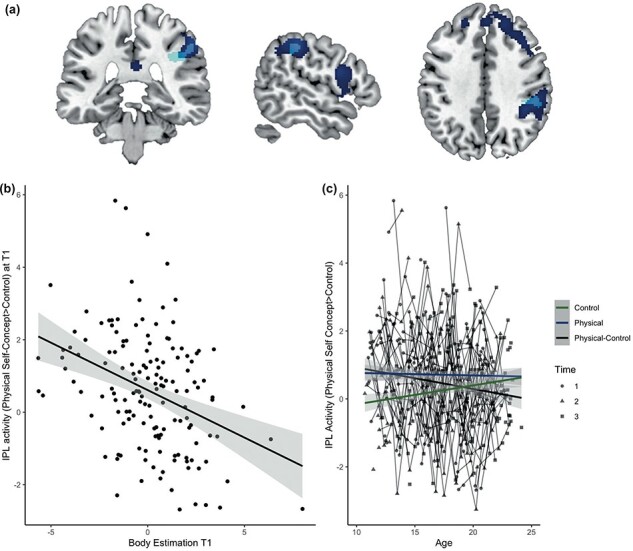
Neural correlates of body image during the physical self-concept task. (a) Brain activation in the physical self-concept > control contrast revealed significantly stronger activation in frontal, parietal, and temporal regions (dark blue) (van der Cruijsen et al. 2018), which showed an overlap with the association between activity and body estimation (light blue) in the IPL. (b) Association between body estimation and IPL activity during the physical self-concept task (in light blue ROI) at T1. (c) Association between IPL activity during the different conditions of the physical self-concept task and age across all timepoints.

Next, the association between body image and neural activation during physical self-concept evaluation based on whole brain regression analyses at the first timepoint (T1) was examined. We performed whole-brain regressions for the contrast Physical self-concept > Control using three measures of body image as regressors [ideal body difference, body dissatisfaction (EDI), and body estimation]. There were no brain regions significantly related to ideal body difference and body dissatisfaction. However, there was an effect of body estimation in the IPL (x = 52, y = –34, z = 42; cluster size = 146) that survived FDR-cluster correction at *P *< .05, in a region overlapping with the network that was observed for the main contrast Physical self-concept > Control (Fig. 7a and Supplemental Fig. S1). For illustration purposes, we extracted the ROI and show the relation with body estimation in Fig. 7b. Follow-up analyses for positive or negative physical self-concept showed associations with body image for both valences (positive > control: *F *= 12.12, *P *< .001; negative > control: *F *= 10.06, *P *= .002). Controlling for age and sex in the ROI analysis did not change the results (*F *= 14.89, *P *< .001).

### Longitudinal association between body image and physical self-concept—fMRI results

Next, the association between body estimation and neural activation during physical self-concept evaluation was examined at the following two timepoints. Activity in the same ROI that showed a significant association with body estimation at T1 was extracted at all timepoints. First, we tested for the developmental trajectory of IPL activity across three timepoints using general linear mixed models with age as quadratic and linear predictor and gender as between-subjects variable. A negative linear association between IPL activity and age was found (*F *= 6.76, *P *= .01), which was the result of an interaction effect of age and condition (Physical and Control) (*F *= 8.01, *P *= .005). To test for an association between body image and neural activity at later timepoints, the analysis was repeated separately per timepoint. However, no relation was found between body estimation and neural activation to physical self-concept at T2 and T3 (*F *= 0.085, *P *= .32; *F *= 0.45, *P *= .50), showing that this relation was specific for the first timepoint. Additionally, the predicting association between neural activity to physical self-concept at T1 and body estimation at T2 and T3 was tested. Neural activity significantly preceded body estimation at T2, but not T3 (*F *= 8.38, *P *= .004; *F *= 0.03, *P *= .86).

Lastly, the association with neural activity in the IPL during the physical self-concept task related to body estimation and changes in body image and physical self-concept was studied. Higher IPL activity at T1 was associated with an increase in body estimation from T1 to T3 (*F *= 8.21, *p_bonferroni_ *= 0.03) but not change in body estimation from T1 to T2 (*F *= 0.45, *p_bonferroni_ *= 0.99). Additionally, IPL activity at T1 was not associated with a change in body dissatisfaction and negative physical self-concept from T1 to T2 and T1 to T3 (*F *= 1.14, *p_bonferroni_ *= 0.99; *F *= 0.58, *p_bonferroni_ *= 0.99; *F *= 0.51, *p_bonferroni_ *= 0.99; *F *= 0.91, *p_bonferroni_ *= 0.99).

## Discussion

In the current study, we aimed to elucidate the developmental trajectory of body image and its neural underpinnings during adolescence. Body image is comprised of several aspects, including an objective, a subjective perceptual (body estimation), and a subjective affective (ideal body difference and body dissatisfaction) component. Body estimation, the alignment between the perceived image of the self and BMI, showed an increase with age, suggesting that with aged adolescents the BMI and perceived image of the self are more aligned. Interestingly, the affective evaluative component, measured as body dissatisfaction, also increased with age, specifically during the pubertal years and it flattened in young adulthood. Body ratings and body dissatisfaction were associated with a less positive and more negative physical self-concept. Body estimation, however, was not associated with behavioral physical self-concept.

When testing for neural correlates during physical self-concept ratings, we found that body estimation was associated with heightened activity in the IPL, meaning that the neural network that is involved with thinking about one’s physical traits is more active for individuals who perceive themselves as larger than they are. The IPL showed continued development during adolescence as it was associated with age, suggesting an interplay between neural development and body perception. Moreover, IPL activity preceded a change in body estimation over the next 2 (but not 1) years, strengthening the conclusion that the IPL in early adolescence is involved in the development of body perception.

### Development of perceptual and affective body-image

The behavioral findings replicate previous work in adolescence that body dissatisfaction and estimation increase throughout adolescence with a flattening toward young adulthood (Eisenberg et al. 2006, Gardner 2012, Wang et al. 2019). While body image showed associations with positive and negative self-concept, the developmental trajectories of body image differ from the development of physical self-concept, which does not show a clear age effect (see also van der Cruijsen et al. 2018). A potential explanation might be that body image is more subjected to the effects of changes of the body during adolescence (i.e. growing and changing shape) than physical self-concept is, which is attested by its stronger relation with BMI (Fernández-Bustos et al. 2019). Moreover, the developmental pattern during adolescence might not just be explained by age, but other individual differences such as pubertal timing might also play a role.

Because of the association between body image and behavioral physical self-concept, we tested whether the neural correlates of physical self-concept are also comprised in body image. The neural results show an association between body estimation and IPL activation such that higher activity was associated with lower body estimating. This means that the IPL is more active for those who perceive their body as larger than they are according to the objective measure. The parietal lobe is involved in the integration of visual information of the body and proprioceptive feedback to assemble body image (Shimada et al. 2005). In people with Anorexia Nervosa, body image therapy affected brain activity in the IPL, confirming its role in body image (Vocks et al. 2010b). We previously showed that the IPL is specific to physical self-concept, compared to academic and prosocial self-concept (van der Cruijsen et al. 2018). Because of its role in the integration of visual information, the IPL has been proposed to play a role in the visual representation of your body: the perceptual component of body image (Gaudio and Quattrocchi 2012). This is in line with the association between IPL activity and body estimation, a perceptual component of body image, and not with body satisfaction, an affective component. The association between body image and IPL activity might suggest that the processing of proprioceptive and visual signals that are involved in body scheme representation affects the body image one creates of oneself (Uher et al. 2005, Hodzic et al. 2009).

Three important findings stand out when interpreting the complex relation between body image and physical self-concept. First, body dissatisfaction showed a positive relation with physical self-concept rating and body estimation showed an association with IPL activity during evaluation of the physical self. Body dissatisfaction was however not associated with IPL activity when evaluating their physical traits and body estimation did not show an association with the behavioral measure of physical self-concept across all timepoints. One explanation is that physical self-concept and body dissatisfaction reflect an affective evaluative component of how one thinks about the self, whereas both body estimation and neural activity in the IPL while thinking about the physical self-concept might reflect a processing, or perceptual, component (Skrzypek et al. 2001, Hodzic et al. 2009). Second, the IPL region that was negatively associated with body estimation showed continued development during adolescent development (see also van der Cruijsen et al. 2023). Specifically, older participants showed less differentiation in neural activity between physical self-concept ratings and control trials. This might suggest that early adolescence is an important time for changing body estimations, as the neural activity to physical self-evaluations shows a larger difference from the activity to control condition relative to older age groups; thus, this heightened IPC activity might contribute to the association with body estimation. Third, we found limited evidence that the neural activity in the IPL at timepoint 1 preceded body image at a later timepoint. Again, this highlights that these results were strongest in early adolescence, which is consistent with other research showing that younger adolescents are, for example, more sensitive to negative mental health effects of social media (Orben and Blakemore 2023).

### Limitations and future directions

The current study has several strengths, such as the inclusion of different aspects of body image measured and the use of a large longitudinal accelerated study to examine the developmental trajectories of body image. Nonetheless, some limitations should also be considered. First, while the relation between body image and neural activity was studied in a relatively large sample of 150 participants, it is crucial for this association to be replicated in independent future studies, especially because the observed relations were limited to the first timepoint. Since neural activity typically shows low intra-individual reliability or high individual variability (Flournoy et al. 2024), replication is highly important. It is especially relevant to test whether the high variability affects the extent to which the fMRI measures can be used for outcome prediction at the individual level. The limited behavioral predictions over time may be the result of low reliability over time, suggesting that fMRI provides better state than trait measures. Second, this study did not examine the effect of the onset of puberty which is known as an important biological marker for body changes. To capture onset of puberty, future studies should include participants aged 8–12 (Farello et al. 2019). Pubertal onset might explain the decrease in body image that we observe throughout adolescence. While results are inconsistent, advanced pubertal status has been associated with more mature functional brain activity, e.g. while reading positive and negative self-descriptions (Pfeifer et al. 2013, Goddings et al. 2019). Third, the rating of the own and ideal body did not show a development with age, which might mean that the figure rating scale is not suitable to capture physical changing bodies, as it is well known that bodies change in physical properties during adolescence. Lastly, future studies should find novel ways to examine the difference between body estimation and ideal body image, e.g. through the use of virtual reality (Irvine et al. 2020).

## Conclusion

Taken together, the results of the present study provide insight into the development of body image and the neural underpinnings of body image during adolescence. These findings highlight the complex interplay between evaluative, perceptual, biological, and affective factors in shaping body image. Moreover, they underscore the need for continued research to better understand and address body image during development, especially in the context of current trends for thin body ideals (Veldhuis et al. 2017). On the one hand, there is currently a large support to embrace body positivity, but, on the other hand, social media norms suggest that a thin body image is preferred among adolescents (Duan et al. 2022, Vandenbosch et al. 2022). Future studies could take these trends into account as peers become increasingly important for adolescents, and they are therefore particularly susceptive for their peers’ opinion (Sebastian et al. 2008, Harter 2012). Studies social-cognitive and social-affective neural responses can inform research on the formation of body image as this is a formative period of changes in developmental processes that might affect body image.

## Supplementary Material

nsae042_Supp

## Data Availability

The data used is the study are available from the corresponding author upon request.
