# Supplementary material for: Developing body estimation in adolescence is associated with neural regions that support self-concept
Source: Soc Cogn Affect Neurosci. 2024 Jun 21;19(1):nsae042. doi: 10.1093/scan/nsae042 (PMC11223914; doi:10.1093/scan/nsae042)
Supplement: nsae042_Supp [file nsae042_supp.zip › scan-23-259-File002.docx]

**Supplemental Material**

Besides the physical self-concept, the original study also examined the neural correlates of academic and prosocial self-concept (van der Cruijsen et al., 2018). In these conditions participants were presented 20 sentences about the academic self-concept (for example: ‘I am motivated at school’) and prosocial self-concept (for example: ‘I take others into account’). To examine the whether the activity in the IPL that was associated with body estimation is specific to the physical self-concept condition, we did a sensitivity analysis. In this analysis, we contrast the ‘physical’ condition with the ‘academic’ and ‘prosocial’ condition as a combined control condition. We found that the relation between body estimation at T1 and IPL activity when evaluating physical traits (contrasted against academic and prosocial traits) trended towards significance (*F*=3.73, *p*=.055) (Supplemental Figure S1).


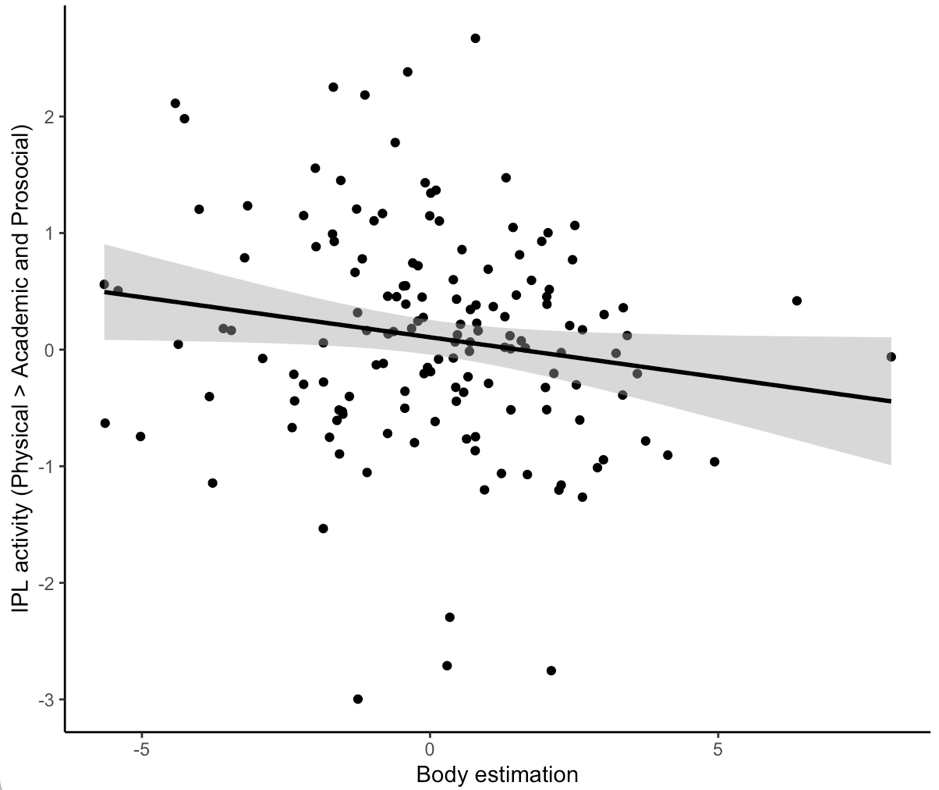


**Figure S1. The association between body estimation and Physical self-concept > Academic and Prosocial self-concept at T1.**

Additionally, the relation between IPL activity (Physical > Control) and age was examined across timepoints. Similar to the results of the IPL activity defined as the region that was associated with body estimation, the larger IPL region (Physical > Control) also showed a negative linear association with age (*F*=9.14, *p*=.003) (Figure S2).


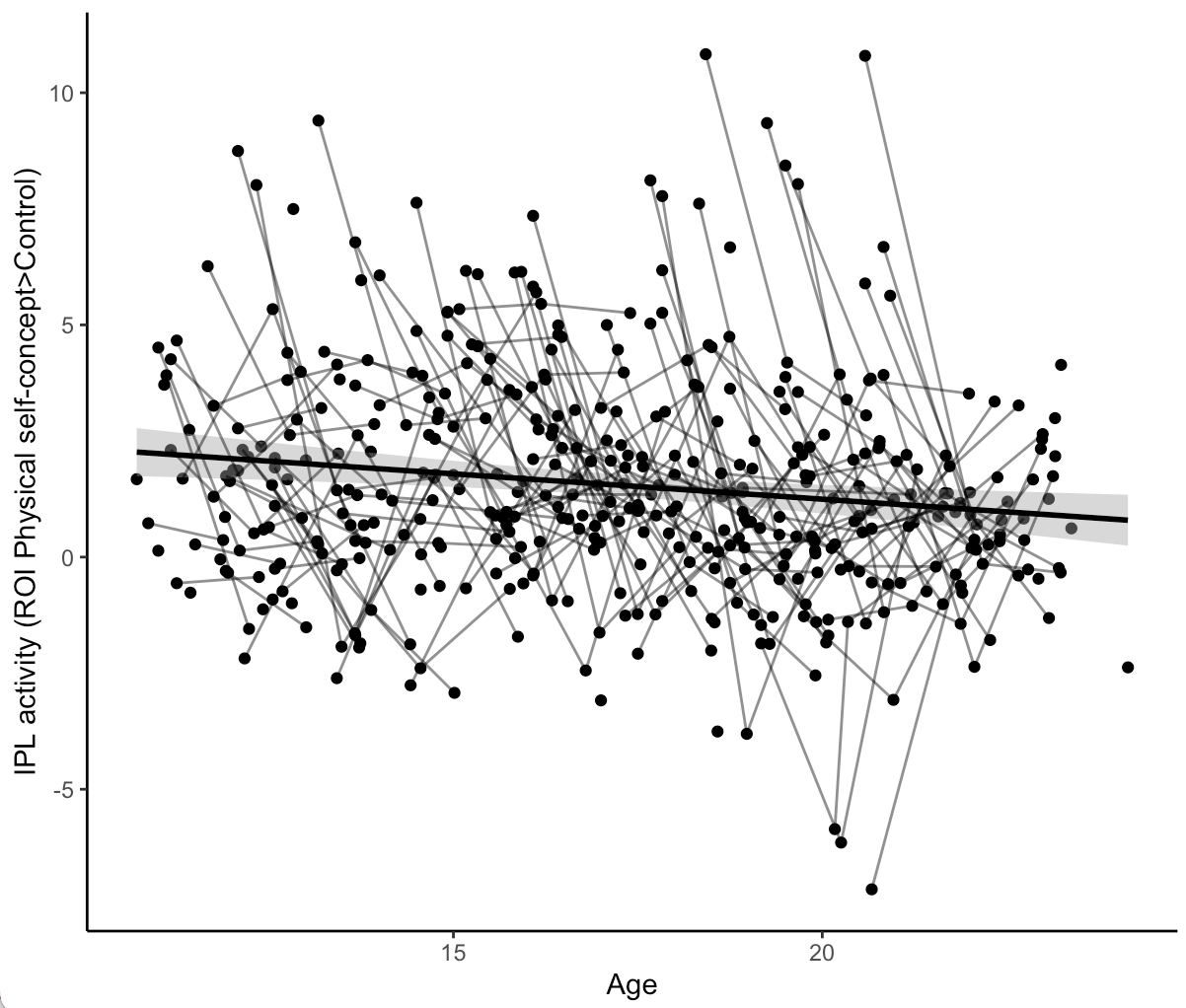


**Figure S2. Relation between IPL activity (defined according to Physical>Control contrast) and age across timepoints.**
